# Cost-effectiveness analysis of the first-line EGFR-TKIs in patients with non-small cell lung cancer harbouring EGFR mutations

**DOI:** 10.1007/s10198-019-01117-3

**Published:** 2019-09-20

**Authors:** Marscha S. Holleman, Maiwenn J. Al, Remziye Zaim, Harry J. M. Groen, Carin A. Uyl-de Groot

**Affiliations:** 1 Erasmus School of Health Policy & Management/Institute for Medical Technology Assessment, Erasmus University Rotterdam, P.O. box 1738, 3000 DR Rotterdam, The Netherlands; 2 Department of Pulmonary Diseases, University of Groningen and University Medical Center Groningen, Groningen, The Netherlands

**Keywords:** Cost-effectiveness analysis, Non-small cell lung cancer, EGFR-TKI, Gefitinib, Erlotinib, Afatinib, Osimertinib, I19, C59, C69

## Abstract

**Objectives:**

To compare the cost-effectiveness of first-line gefitinib, erlotinib, afatinib, and osimertinib in patients with non-small cell lung cancer (NSCLC) harbouring epidermal growth factor receptor (EGFR) mutations.

**Methods:**

A systematic review and network meta-analysis (NMA) were conducted to compare the relative efficacy of gefitinib, erlotinib, afatinib, and osimertinib in EGFR-mutated NSCLC. To assess the cost-effectiveness of these treatments, a Markov model was developed from Dutch societal perspective. The model was based on the clinical studies included in the NMA. Incremental costs per life-year (LY) and per quality-adjusted life-year (QALY) gained were estimated. Deterministic and probabilistic sensitivity analyses (PSA) were conducted.

**Results:**

Total discounted per patient costs for gefitinib, erlotinib, afatinib, and osimertinib were €65,889, €64,035, €69,418, and €131,997, and mean QALYs were 1.36, 1.39, 1.52, and 2.01 per patient, respectively. Erlotinib dominated gefitinib. Afatinib versus erlotinib yielded incremental costs of €27,058/LY and €41,504/QALY gained. Osimertinib resulted in €91,726/LY and €128,343/QALY gained compared to afatinib. PSA showed that gefitinib, erlotinib, afatinib, and osimertinib had 13%, 19%, 43%, and 26% probability to be cost-effective at a threshold of €80,000/QALY. A price reduction of osimertinib of 30% is required for osimertinib to be cost-effective at a threshold of €80,000/QALY.

**Conclusions:**

Osimertinib has a better effectiveness compared to all other TKIs. However, at a Dutch threshold of €80,000/QALY, osimertinib appears not to be cost-effective.

**Electronic supplementary material:**

The online version of this article (10.1007/s10198-019-01117-3) contains supplementary material, which is available to authorized users.

## Introduction

Lung cancer is the leading cause of cancer-related mortality in The Netherlands and worldwide, with 10,346 lung cancer deaths in The Netherlands in 2014 [1]. Non-small cell lung cancer (NSCLC) is the most common type of lung cancer with 80–85% of all cases [2]. At diagnosis, many patients with NSCLC are already in an advanced disease stage (IIIB or IV) and thus ineligible for surgical resection [3]. Platinum-based therapy is the standard first-line treatment for advanced NSCLC, which provides a median overall survival (OS) of about 8 months [4]. Nowadays, molecularly targeted agents are of high importance as treatment strategies for lung cancer patients [5]. For several cancer types, these targeted agents have come with improved outcomes, but also increased costs [6].

In NSCLC, mutations of the epidermal growth factor receptor (EGFR) play an important role in the growth and progression of tumour cells [7]. Prevalence of EGFR mutations is the highest in Asia with over 50% of all Asian patients with lung cancer type adenocarcinoma [8]. Among Dutch patients with NSCLC, the frequency of EGFR mutations is about 11% [9, 10]. Currently, three first-line EGFR tyrosine kinase inhibitors (TKIs) are used in clinical practice: gefitinib, erlotinib, and afatinib. These drugs have shown significantly improved progression-free survival (PFS) as the first-line treatment, compared to platinum-based therapy, in patients with EGFR mutation-positive (exon 19 deletion or exon 21 L858R mutation) NSCLC [11–18]. Osimertinib, a third-generation EGFR-TKI, is used as the second-line treatment in clinical practice. Recently, a randomised-controlled trial (RCT) showed a better efficacy of osimertinib compared to gefitinib and erlotinib as the first-line treatment. Moreover, clinical studies showed the ability of osimertinib to penetrate the central nervous system (CNS). This may be an advantage over the standard treatment, as it could decrease the occurrence of CNS progression [19]. Therefore, osimertinib is expected to be used as the first-line treatment in clinical practice in the near future. Clear direct evidence of the differences between gefitinib, erlotinib, afatinib, and osimertinib in terms of efficacy and toxicity is lacking as head-to-head comparisons are not available for all these TKIs. Thus, it is still uncertain whether one TKI is more favourable over the others in terms of efficacy and toxicity. Network meta-analysis (NMA) enables comparison of direct and indirect evidence across trials to synthesise the efficacy of different TKIs. Several NMAs on TKIs did not show significant differences between these drugs [20–24]. However, the outcomes of the NMAs differed from each other, which may be due to differences in the selection of studies and data [25]. Therefore, we built a new NMA of the efficacy of first-line gefitinib, erlotinib, afatinib, and osimertinib. In addition, lung cancer has a substantial economic burden on the health care system, with total mean hospital costs of €33,143 per patient with NSCLC in The Netherlands [26]. For NSCLC, furthermore, TKIs are administered until disease progression or unacceptable toxicity, which increases the drug acquisition costs. Nowadays, the comparative costs and effects are of growing importance for decision-makers [27]. Therefore, information on the incremental value of new treatments in terms of effects and costs is needed for medical resource optimisation. However, not only the acquisition costs of the drugs should be taken into account in the assessment of the cost-effectiveness, but also, for example, costs of adverse event management, travelling, and productivity losses [28]. Hence, we aimed to assess the cost-effectiveness of first-line gefitinib, erlotinib, afatinib, and osimertinib in patients with stage IIIB/IV NSCLC harbouring EGFR mutations (exon 19 deletion or exon 21 L858R mutation) in The Netherlands from a Dutch societal perspective.

## Methods

### Systematic review and network meta-analysis

A systematic search of several databases (PubMed, EMBASE, and Cochrane Library) was conducted to identify phase IIB/III RCTs of first-line EGFR-TKI (including gefitinib, erlotinib, afatinib, or osimertinib) compared to another TKI or platinum-based therapy. Search strategy and inclusion and exclusion criteria can be found in Appendix I. Reference lists of published studies were also checked as additional information. The literature review was conducted by two reviewers (MH and CU). After screening titles and abstracts and then full-text reading of the records found by the systematic review, 12 unique RCTs were included in the NMA [11–17, 19, 29–32]. Quality and risk of bias of the included RCTs were assessed using the Cochrane Collaboration’s tool for assessing risk of bias. According to this assessment, all RCTs were classified as having acceptable quality and low risk of bias [25]. Data on patient characteristics, interventions, comparators, and treatment effects [PFS, OS, and adverse events (AEs)] were extracted. For the NMA, the outcomes of interest were PFS and OS. Since no separate HRs of osimertinib versus gefitinib or osimertinib versus erlotinib were reported in the FLAURA trial, the HRs of PFS and OS were assumed to be the same for both comparisons. A fixed-effects network meta-analysis in WinBUGS 1.4 was built within a Bayesian framework by use of an adapted version of WinBUGS code from Dias et al. [33]. Due to the limited number of RCTs per TKI arm, heterogeneity could not be appropriately assessed. Therefore, a fixed-effect NMA was considered as appropriate. The methods of the NMA are described in more detail in Appendix I and in a previous study [34]. The results of the NMA are presented in Table 1. Osimertinib had a significantly better PFS and OS compared to gefitinib, erlotinib, and afatinib.

**Table 1:** NMA results of PFS and OS

PFS				
Chemotherapy	2.34 (2.04, 2.71)	2.76 (2.3, 3.34)	2.70 (2.27, 3.24)	5.63 (4.58, 7.01)
0.43 (0.37, 0.49)	Gefitinib	1.17 (0.98, 1.41)	1.15 (0.96, 1.39)	2.40 (2, 2.90)
0.36 (0.3, 0.44)	0.85 (0.71, 1.02)	Erlotinib	0.97 (0.77, 1.24)	2.04 (1.7, 2.46)
0.37 (0.31, 0.44)	0.87 (0.72, 1.04)	1.03 (0.8, 1.3)	Afatinib	2.07 (1.62, 2.69)
0.18 (0.14, 0.22)	0.42 (0.34, 0.5)	0.49 (0.41, 0.59)	0.48 (0.37, 0.62)	Osimertinib
OS				
Chemotherapy	0.97 (0.84, 1.12)	0.99 (0.83, 1.19)	1.11 (0.94, 1.31)	1.54 (1.19, 2.04)
1.03 (0.89, 1.19)	Gefitinib	1.02 (0.84, 1.24)	1.14 (0.96, 1.38)	1.59 (1.24, 2.07)
1.01 (0.84, 1.21)	0.98 (0.80, 1.19)	Erlotinib	1.11 (0.89, 1.41)	1.56 (1.22, 2.03)
0.90 (0.76, 1.06)	0.88 (0.73, 1.05)	0.90 (0.7, 1.13)	Afatinib	1.38 (1.04, 1.89)
0.65 (0.49, 0.84)	0.63 (0.48, 0.81)	0.64 (0.49, 0.82)	0.72 (0.53, 0.96)	Osimertinib

*PFS* progression-free survival, *OS* overall survival

### Model construction

A Markov model was constructed simulating the transition between three health states: progression-free, progression, and death, in which death was an absorbing state. A cycle length of 30 days was used for the model, which is an appropriate length given the rate at which lung cancer develops. In this model, during each cycle, patients with EGFR-mutated NSCLC move between the health states according to the transition probabilities. In each cycle, patients could remain progression-free, may progress, or die. A lifetime time horizon was used, in line with the Dutch guidelines [28], accounting for all relevant costs and effects of TKI therapies for patients with EGFR mutations. Half-cycle correction was applied to both costs and effects. Effects are expressed in life-years (LYs) gained and in quality-adjusted life-years (QALYs) gained. Outcomes are presented as incremental cost-effectiveness ratios (ICERs), i.e., incremental costs per LY gained and incremental costs per QALY gained.

### Clinical effectiveness

Estimates of the clinical effectiveness in terms of pooled HRs were derived from the NMA. Since HRs only convey information on comparative effectiveness, whereas a model requires absolute estimates of PFS and OS, we used an indirect approach to estimate the transitions of patients treated with TKIs in the model. The NMA did not only include the four TKIs, but also chemotherapy. Thus, we first explored the Kaplan–Meier (KM) curves of PFS and OS for patients with EGFR mutations treated with chemotherapy from the EURTAC trial of erlotinib versus chemotherapy. According to clinical experts, the data of the chemotherapy patients in the EURTAC trial [15] were deemed as most representative for our study as patient characteristics of that trial are most similar to the Dutch patient population eligible for TKIs (i.e., Caucasian population, mainly adenocarcinoma histology, mainly stage IV NSCLC). However, as the time horizon of the model is life time, whereas the KM curves are truncated at 40 months, where 15% of the patients are still alive, it was necessary to extrapolate the KM curve using a parametric survival curve. Since we had no access to the individual patient data (IPD) of the EURTAC trial, the method of Hoyle and Henley [35] was used to recreate the IPD. Times and survival probabilities were read off from the published KM graph. Based on these survival probabilities and corresponding time and provided numbers at risk, the method of Hoyle and Henley estimated the underlying number of events and censorships in each time interval. By use of the statistical programme R, several survival distributions were fit to the recreated IPD. Based on the fit to the KM curve and the Akaike and Bayesian Information Criterion (AIC and BIC) estimates, a Weibull distribution was assessed as having the best goodness-of-fit for both PFS and OS (see Appendix II). The general Weibull equation is as follows (in which ‘*t*’ is time in months): $S ( t ) = e^{ - \lambda t^{\gamma } }.$ Lambda and gamma parameters of the patients treated with chemotherapy in the EURTAC trial were used to estimate the parameters for gefitinib, erlotinib, afatinib, and osimertinib, as previously described in published studies [36, 37]. For example, the lambda parameter (scale parameter) for gefitinib was estimated by multiplying the lambda for chemotherapy by the pooled HR of gefitinib versus chemotherapy. The gamma parameter (shape parameter) was set equal to the gamma for chemotherapy. The same was done for erlotinib, afatinib, and osimertinib. These parameters were used as input to calculate the transitions of all TKIs.

For each TKI, the percentage of patients in progression-free state at each time is determined by the values of the PFS curve at that time. Similarly, the percentage of patients in the death state is determined as 1 minus the OS curve at that time. From this, the percentage of patients in the progressed state follows, as the three states together should always add up to 100%.

After progression on first-line gefitinib, erlotinib, or afatinib, patients were tested for T790 M mutations. Patients who were T790 M mutation-positive received the second-line osimertinib (50% of all patients) and patients who were T790 M mutation-negative were treated with pemetrexed–cisplatin [5, 38]. Patients who had progressive disease on the first-line osimertinib received the second-line pemetrexed–cisplatin treatment. Thus, the progressed health state is split into a ‘progression-free second line’ and ‘progressed second line’ health state for those patients receiving a second-line treatment. Clinical data of second-line osimertinib and pemetrexed–cisplatin were derived from the literature [39, 40]. The KM curves of second-line osimertinib and pemetrexed–cisplatin were also extrapolated by fitting various parametric functions. For both second-line PFS and OS, the exponential function was assessed as having the best fit to the KM curves of second-line osimertinib and pemetrexed–cisplatin. The survival curves of all treatment options and the estimation of the transition parameters can be found in Appendix II. After progression on the second-line osimertinib or pemetrexed-cisplatin, it was assumed that patients were treated with best supportive care (BSC) until death.

### Utility weights

Health utility values reflecting the health-related quality of life in each health state were obtained from the literature [41]. The progression-free health state had the highest possible utility value while receiving TKI, with an estimated value of 0.71. This utility value was the same for all three TKI treatments. Progressive disease led to disutility for all TKIs. After progression on the first-line TKI treatment, the utility value was estimated at 0.67 (irrespective of post-progression treatment with osimertinib or pemetrexed–cisplatin) and after progression on the second-line treatment at 0.62 [41].

Disutility scores of severe adverse events (SAEs) with grades 3 or higher for the first-line gefitinib, erlotinib, afatinib, osimertinib, second-line osimertinib, and pemetrexed-cisplatin were also included in the analyses. Occurrence of SAEs was extracted from the RCTs [11–19, 30–32] and were only included when at least 1.5% of the patients experienced a certain SAE. The disutility estimates were derived from the literature. The SAEs were assumed to all occur in the first simulation cycle of that specific treatment, since the adverse events commonly appear within the first weeks after starting these treatments [42, 43]. For the future effects, a discount rate of 1.5% was applied, according to the Dutch guidelines [28]. All utility values are presented in Table 2.

**Table 2:** Input parameters for the model

	Base case	Input DSA	Distribution	References
Costs				
Gefitinib per cycle	€2526^a^		Gamma	[27]
Erlotinib per cycle	€2260^a^		Gamma	[27]
Afatinib per cycle	€2414^a^		Gamma	[27]
Osimertinib per cycle	€6106^a^		Gamma	[43]
Pemetrexed/cisplatin per cycle^b^	€3029^a^		Gamma	[27]
Best supportive care per cycle	€1775	1377; 2065^k^	Gamma	[44]
Mutation test	€929	604; 906^k^	Gamma	[45]
Tumour response assessment^c^	€405	157; 236^k^	Gamma	[45]
Outpatient visit	€83	65; 97^k^	Gamma	[46]
Laboratory tests^d^	€77	60; 89^k^	Gamma	[26]
Drug administration	€271	210; 315^k^	Gamma	[44]
CNS progression osimertinib	€535	428; 642	Gamma	[47]
CNS progression standard-TKI	€1250	1000; 1500	Gamma	[47]
End-of-life	€2196	1703; 2555^k^	Gamma	[48]
Home care per hour	€11	9;13 ^k^	Gamma	[49]
Indirect medical costs	€10,602^l^	4578; 26,326	Gamma	[50]
Informal care per hour	€14	11; 17^k^	Gamma	[49]
Travelling	€6^e^	5; 7^k^	Gamma	[46]
Productivity loss	€4068	3155; 4733^k^	Gamma	[51]
ALT/AST increase	€464	360; 540^k^	Gamma	[27]
Anaemia	€1953	1514; 2272^k^	Gamma	[49]
Anorexia	€797	618; 927^k^	Gamma	[52]
Asthenia	€813	631; 946^f,k^	Gamma	[49]
Decreased appetite	€826	640; 961^k^	Gamma	[49]
Decreased white blood cells	€1405	1089; 1634^g,k^	Gamma	[49]
Diarrhoea	€2359	1830; 2744^k^	Gamma	[49]
Dyspnoea	€467	362; 543^k^	Gamma	[27]
Fatigue	€813	631; 946^k^	Gamma	[49]
Febrile neutropenia	€3033	2353; 3,529^k^	Gamma	[49]
Leukopenia	€1942	1507; 2260^k^	Gamma	[49]
Nausea	€728	565; 847^k^	Gamma	[49]
Neuropathy	€795	616; 924^k^	Gamma	[49]
Neutropenia	€1405	1089; 1634^k^	Gamma	[49]
Paronychia	€2359^j^	1830; 2744^k^	Gamma	[49]
Rash	€2359	1830; 2744^k^	Gamma	[49]
Stomatitis	€4229	3280; 4920^k^	Gamma	[53]
Vomiting	€728^i^	565; 847^k^	Gamma	[49]
Utilities				
Progression-free	0.71	0.67; 0.80	Beta	[40]
After progression	0.67	0.59; 0.75	Beta	[40]
After progression on second line	0.62	0.49; 0.74	Beta	[40]
Disutilities				
ALT/AST increase	− 0	0; 0^k^	Beta	[54]
Anaemia	− 0.125	− 0.10; − 0.15^k^	Beta	[49]
Anorexia	− 0.142	− 0.114; − 0.170	Beta	[55]
Asthenia	− 0.074^f^	− 0.037; − 0.110	Beta	[56]
Decreased appetite	− 0.048	− 0.016; − 0.080	Beta	[49]
Decreased white blood cells	− 0.090^g^	− 0.060; − 0.120	Beta	[56]
Diarrhoea	− 0.047	− 0.016; − 0.078	Beta	[56]
Dyspnoea	− 0.256	− 0.204; − 0.307^k^	Beta	[55]
Fatigue	− 0.074	− 0.037; − 0.110	Beta	[56]
Febrile neutropenia	− 0.090	− 0.058; − 0.122	Beta	[49]
Leukopenia	− 0.090	− 0.059; − 0.120	Beta	[49]
Nausea	− 0.048	− 0.016; − 0.080	Beta	[56]
Neuropathy	− 0.048	− 0.016; − 0.080	Beta	[49]
Neutropenia	− 0.090	− 0.060; − 0.120	Beta	[56]
Paronychia	− 0.033^j^	− 0.009; − 0.056	Beta	[56]
Rash	− 0.033	− 0.009; − 0.056	Beta	[56]
Stomatitis	− 0.151	− 0.121; − 0.181^k^	Beta	[57]
Vomiting	− 0.048	− 0.016; − 0.080	Beta	[56]
Body surface area	1.70	1.36; 2.04	Normal	[49]
Parameters survival distribution				
Lambda OS chemotherapy	0.019		Normal	
Gamma OS chemotherapy	1.203		Normal	
Lambda OS gefitinib	0.020		Normal	
Gamma OS gefitinib	1.203		Normal	
Lambda OS erlotinib	0.019		Normal	
Gamma OS erlotinib	1.203		Normal	
Lambda OS afatinib	0.017		Normal	
Gamma OS afatinib	1.203		Normal	
Lambda OS osimertinib	0.012		Normal	
Gamma OS osimertinib	1.203		Normal	
Intercept OS second-line osimertinib	4.069		Normal	
Intercept OS second-line pemetrexed/cisplatin	2.861		Normal	
Lambda PFS chemotherapy	0.073		Normal	
Gamma PFS chemotherapy	1.478		Normal	
Lambda PFS gefitinib	0.031		Normal	
Gamma PFS gefitinib	1.478		Normal	
Lambda PFS erlotinib	0.026		Normal	
Gamma PFS erlotinib	1.478		Normal	
Lambda PFS afatinib	0.027		Normal	
Gamma PFS afatinib	1.478		Normal	
Lambda PFS osimertinib	0.013		Normal	
Gamma PFS osimertinib	1.478		Normal	
Intercept PFS second-line osimertinib	2.985		Normal	
Intercept PFS second-line pemetrexed/cisplatin	1.885		Normal	

*CNS* central nervous system, *DSA* deterministic sensitivity analysis, *OS* overall survival, *PFS* progression-free survival, *ALT* alanine aminotransferase; *AST* aspartate aminotransferase

^a^Costs comprised of acquisition costs and pharmaceutical delivery costs; no drug wastage assumed

^b^Volume pemetrexed/cisplatin based on a point estimate body surface of 1.70 m^2^. Administration of 500 mg/m^2^ pemetrexed and 75 mg/m^2^ cisplatin each cycle

^c^Tumour response assessment comprised CT and MRI scans for tumour assessment

^d^Laboratory costs comprised haematology, sputum, and biochemistry test, excluding mutation test

^e^Based on 14 km (€0.19/km) plus parking costs (€3, –)

^f^Assumed to be the same as fatigue

^g^Assumed to be the same as neutropenia

^h^Assumed to be the same rash

^i^Assumed to be the same as nausea

^j^Assumed to be the same as rash

^k^Parameters were varied with ± 20% of the mean

^l^€10,602 are the average indirect medical costs over a lifetime horizon. Indirect medical costs ranged between €4578 and €26,326

### Costs

Following the Dutch guideline, a societal perspective was used for the model. Table 2 shows all unit costs of gefitinib, erlotinib, afatinib, and osimertinib treatment. Costs were based on the Dutch Costing manual, the Dutch Health Care Institute, Dutch Healthcare Authority, and the literature [27, 44, 45]. All costs are in Euros, based on the average consumer price index of 2018. Future costs were discounted by a rate of 4%, according to the Dutch guidelines [28]. More details on the costs can be found in Appendix II.

### Sensitivity analyses

Since the cost-effectiveness model is based on a number of assumptions, several scenario analyses were performed to test the robustness of these assumptions. In the first scenario tested, a log-logistic function instead of the Weibull function was used to estimate the survival probabilities in the model. Second, the chemotherapy patient group from another clinical trial (Lux-Lung 6) [17] was used to estimate the survival probabilities of gefitinib, erlotinib, afatinib, and osimertinib. Third, docetaxel instead of pemetrexed–cisplatin was included as the second-line treatment.

Deterministic (DSA) sensitivity analysis was performed to determine which input parameters of the model were most influential on the results of the model and to test the robustness of the model. In this DSA, the impact of varying single input parameters on the cost-effectiveness ratio while holding the others constant was assessed. If available, the 95% confidence intervals (CI) of input estimates were used for the DSA. If not, parameters were varied with ± 20% of the mean. Probabilistic sensitivity analysis (PSA) was performed by simultaneously varying all the input parameters in a Monte Carlo simulation according to pre-specified distributions. Survival parameters lambda and gamma were assumed to be bivariate normal distributed, for utilities and probabilities, a beta distribution was applied and a gamma distribution was used for costs. Standard errors of utilities and probabilities were either obtained from the literature or calculated by 10% of the mean point estimate and 20% was used for the costs. In total, 1000 simulation samples were randomly drawn from the distributions of all inputs, and each time, the model results (incremental costs and incremental effects) were recalculated. We constructed a cost-effectiveness plane that shows the base-case ICER and the uncertainty surrounding the estimated costs and effects of the pairwise comparisons. Based on the cost-effectiveness plane, a cost-effectiveness acceptability curve was constructed, which shows the probability that a treatment is cost-effective compared to the alternative, given a range of threshold ICERs [46, 47].

## Results

### Base-case results

Table 3 shows the incremental base-case results of the cost-effectiveness analyses. Gefitinib and erlotinib showed the lowest total discounted costs per patient and osimertinib had the highest estimated costs for patients with EGFR-mutated NSCLC. Osimertinib yielded the most effects, followed by afatinib, erlotinib, and gefitinib. Compared to gefitinib, erlotinib resulted in a QALY gain of 0.03 (and 0.03 LYs) and cost savings of €1854 per patient, indicating that erlotinib dominates gefitinib. Afatinib compared to erlotinib yielded 0.13 QALYs (and 0.20 LYs) gained and a cost increase of €5383 per patient, which resulted in an ICER of €27,058/LY and €41,504/QALY for afatinib versus erlotinib. Osimertinib yielded 0.49 QALYs (and 0.68 LYs) and €62,579 more costs relative to afatinib. Thus, an additional €91,726 per LY and €128,343 per QALY gained is spent on osimertinib compared to afatinib. The results of all other comparisons can be found in Appendix III.

**Table 3:** Base-case results of cost-effectiveness analyses

Comparison	Costs (€)	Costs 1st-line (€)	LYs	QALYs	Δ Costs (€)	Δ Effects	ICER (€)
Gefitinib	65,889	39,467	2.01	1.36	–	–	–
Erlotinib	64,035	39,825	2.04	1.39			Dominates gefitinib
Afatinib	69,418	42,416	2.24	1.52	5383	0.13	41,504
Osimertinib	131,997	124,149	2.92	2.01	62,579	0.49	128,343

*ICER* incremental cost-effectiveness ratio, *LYs* life-years, *QALYs* quality-adjusted life-years, *Δ* difference in costs/effects

### Scenario analysis

Considering a Dutch threshold of €80,000/QALY, osimertinib appears not to be cost-effective (ICER of osimertinib vs. afatinib was €128,343/QALY). For osimertinib, a price reduction of 30% is required to be regarded as cost-effective (Appendix III).

### Sensitivity analyses

Based on visual inspection, the Log-Logistic distribution for PFS can be regarded as a plausible alternative for the Weibull distribution. Since the Log-Logistic distribution also scored second for AIC and BIC (see Appendix II), we performed a scenario analysis using the Log-Logistic distribution to estimate the survival probabilities, which were then included into the model. This mainly resulted into lower incremental costs and a lower ICER for osimertinib compared to afatinib. In another scenario, the chemotherapy patient group from the Lux-Lung 6 trial [17] was used instead of the EURTAC trial to estimate the survival probabilities of the TKIs. This scenario resulted in lower incremental costs and QALYs, especially for the comparison of osimertinib versus afatinib. Inclusion of another second-line treatment than pemetrexed-cisplatin hardly affected the results (see Table C2 in Appendix III).

**Fig. 1:**
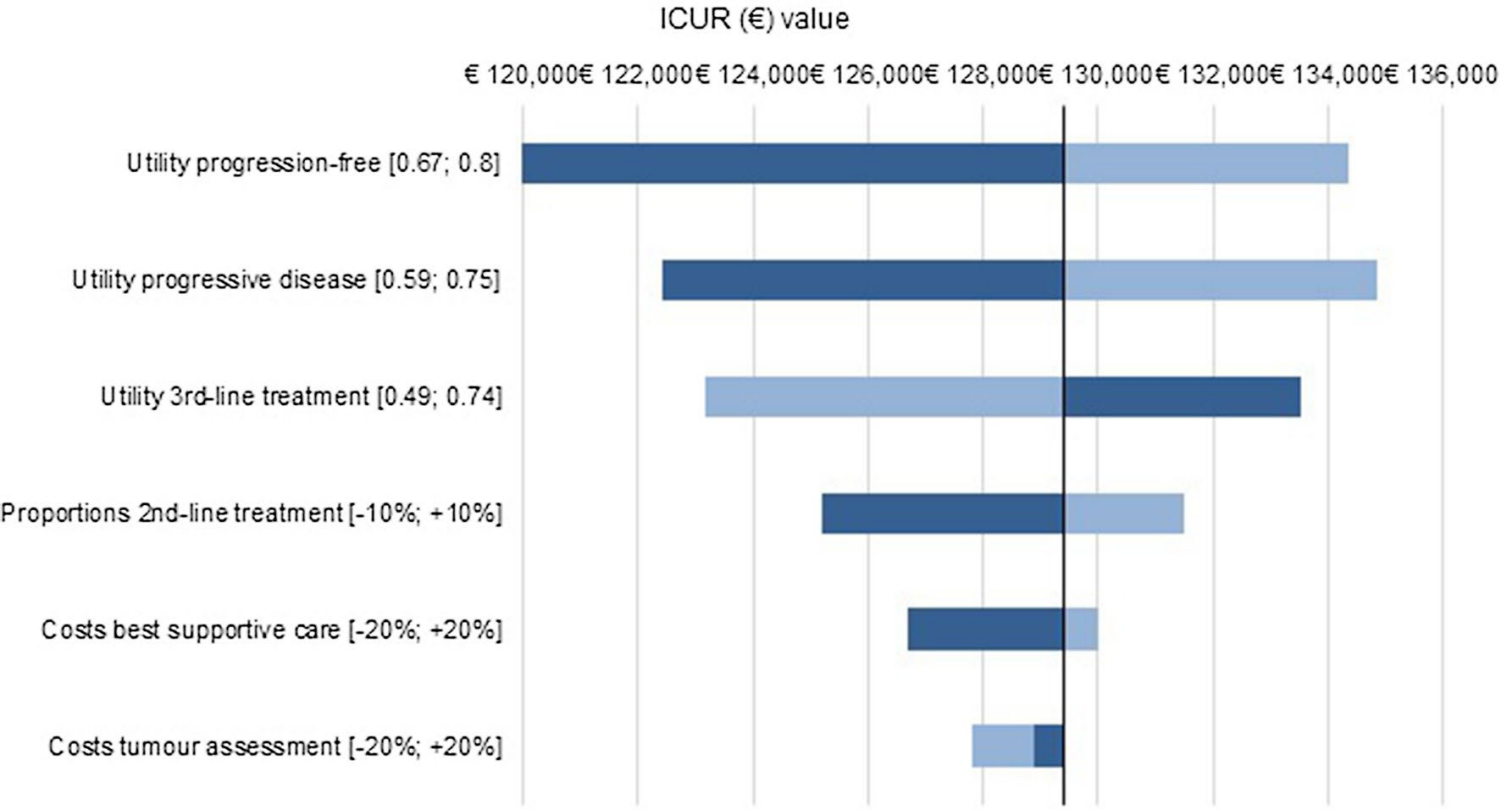
Base-case Tornado diagram of the ICER of osimertinib vs. afatinib. *ICER* incremental cost-effectiveness ratio

Since the comparison of osimertinib versus afatinib is most interesting (as gefitinib is dominated by erlotinib and afatinib is cost-effective compared to erlotinib), only the tornado diagram of this comparison is presented here (Fig. 1). DSA showed that the utility value of the progression-free health state seemed to be the most influential drivers. The tornado diagrams of erlotinib versus gefitinib and of afatinib versus erlotinib can be found in Appendix III.

**Fig. 2:**
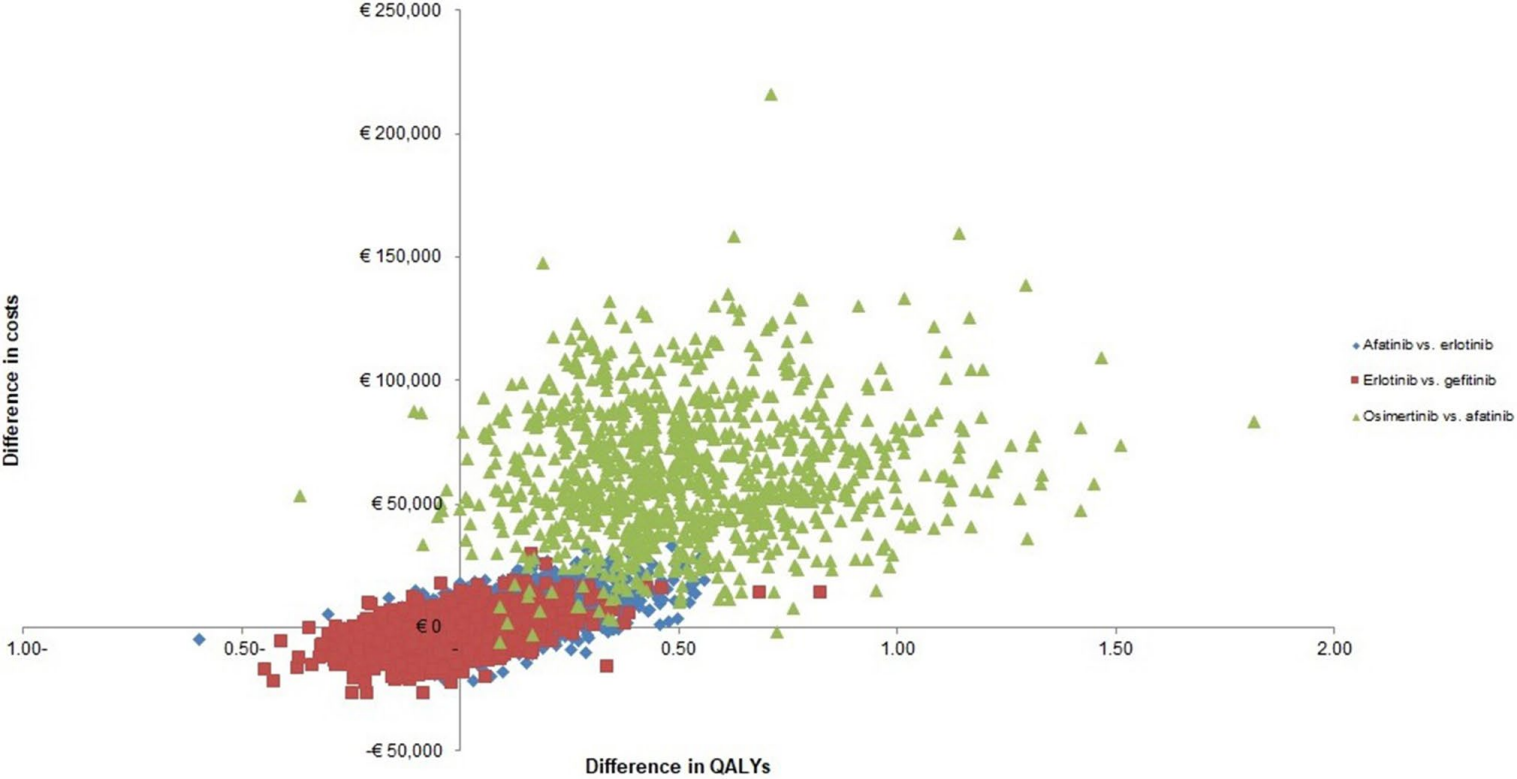
Cost-effectiveness plane of all comparisons. *QALYs* quality-adjusted life-years

**Fig. 3:**
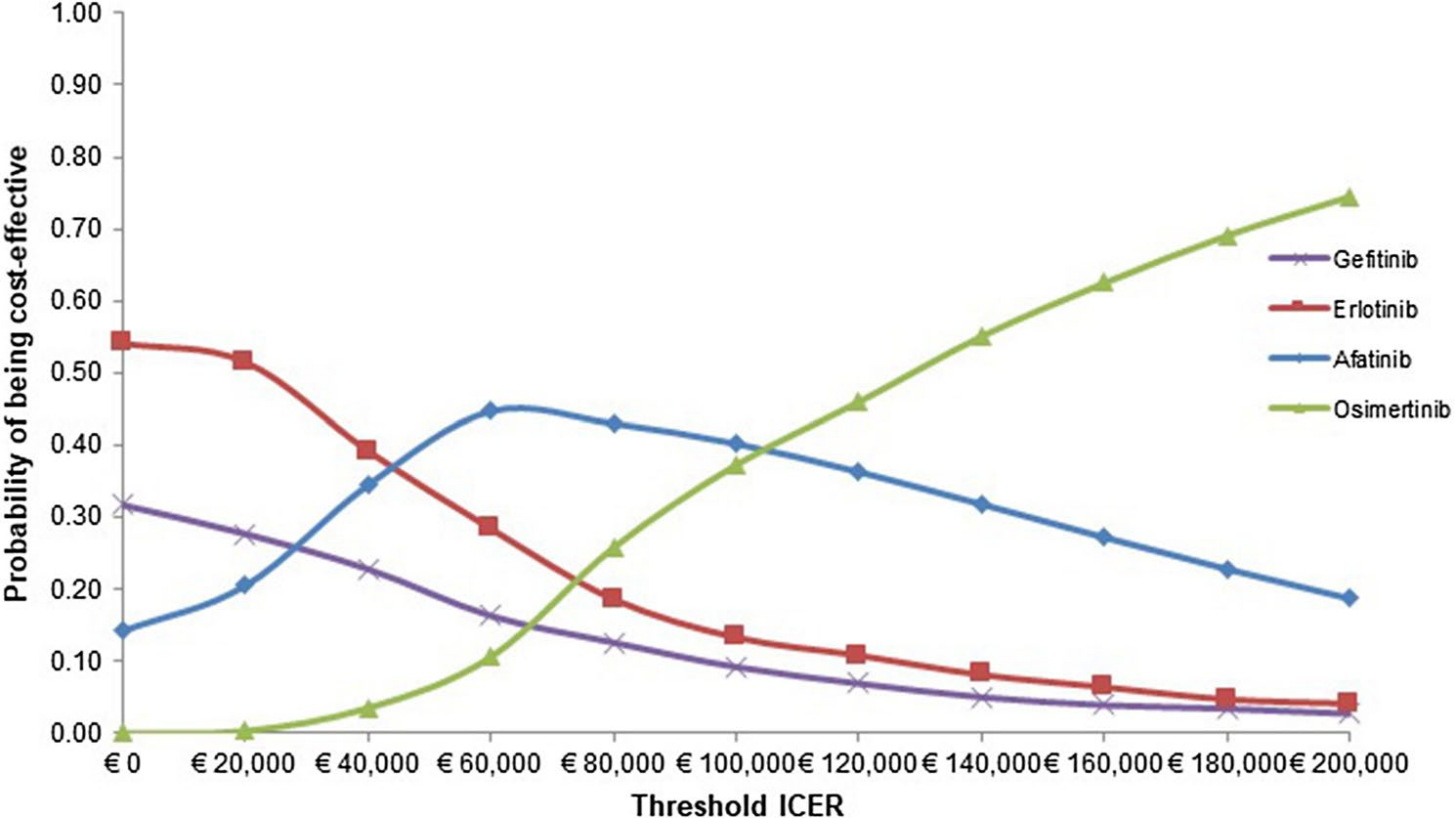
Cost-effectiveness acceptability curves. *ICER* incremental cost-effectiveness ratio

Figure 2 shows that almost 100% of the 1000 PSA iterations were in the upper right quadrant, which means that more QALYs gained at additional costs for osimertinib compared to afatinib. For afatinib versus erlotinib, about 60% of the PSA iterations were in the upper right quadrant, 20% fell within the lower right quadrant, 10% in the upper left, and another 10% was in the lower left quadrant. For erlotinib compared to gefitinib, about 30% of the iterations fell within both the lower left and upper right quadrant, and about 20% fell within both the upper left and lower right quadrant. The cost-effectiveness acceptability curves (CEAC) of all TKIs are shown in Fig. 3. At a Dutch threshold of €80,000/QALY, afatinib had the highest probability of being cost-effective (43%). Gefitinib, erlotinib, and osimertinib had a probability of 13%, 19%, and 26%, respectively, of being cost-effective at the Dutch threshold. At a threshold of €200,000/QALY, the probability of being cost-effective was 75% for osimertinib.

## Discussion

To the best of our knowledge, this was the first study in The Netherlands that compared the cost-effectiveness of first-line gefitinib, erlotinib, afatinib, and osimertinib for EGFR mutation-positive (exon 19 deletion or exon 21 L858R mutation) NSCLC patients. Our study found that erlotinib dominated gefitinib. Afatinib resulted in a cost per QALY of €41,504 compared to erlotinib. Compared to afatinib treatment, osimertinib had an ICER of €128,343 per QALY gained. Thus, osimertinib was the most efficacious treatment option, followed by afatinib, erlotinib, and gefitinib, but at a high cost.

Our results are similar to the results of Aguiar et al. with ICERs of $219,874/QALY of osimertinib vs. afatinib in the US and $175,432/QALY in Brazil [48]. In a report from the Dutch Health Care Institute (ZIN), osimertinib yielded an ICER of €324,006/QALY compared to gefitinib, erlotinib, and afatinib. An ICER range from €70,847 to €324,006 was reported and the upper limit was used to calculate the required price reduction for osimerinib to be regarded as cost-effective (reduction of 55% at threshold of €80,000). The study submitted to ZIN used the effectiveness of only one trial (FLAURA trial), and thus, not all available evidence was used to estimate the effectiveness of the drugs. Utility values for progression-free health state also differed: 0.829 in the report versus 0.71 in this study [41, 49]. Since the utility values reported by the manufacturer were higher than previous reported utility values for this patient population, these values were not used in this study. When we take these aspects into account, our results would be in the order of the findings of the ZIN report. In other cost-effectiveness studies, only two TKIs were compared [50–53]. Lee et al. [51] showed incremental costs per QALY gained by erlotinib compared to gefitinib of $62,419 (incremental costs $14,061 and incremental QALY 0.23) and $41,494 per LY gained (incremental LY 0.34). These results are different from our study. This might be due to the fact that Lee et al. [51] simulated the survival probability for erlotinib based on the OS outcomes of the IPASS trial [18], because the OS results of erlotinib were still immature at that moment. In addition, more studies were included in our analyses. Ting et al. [50] analysed the cost-effectiveness of erlotinib versus afatinib and found a mean ICER of $61,809/QALY, with incremental costs $6417 and incremental QALY 0.17 [50]. These outcomes are the opposite of our results. A plausible reason might be that only the EURTAC and Lux-Lung 3 trials were used for the data of erlotinib and afatinib, while we included various trials besides these two in our network [11–17, 19, 29–32]. Furthermore, Ting et al. [50] have corrected the survival probabilities of erlotinib for patients with more severe disease. However, survival estimates were not corrected for other prognostic factors that were unequally distributed among the two treatments (e.g., EGFR mutation type). Correcting for only one prognostic factor could result into biased corrections. When uncorrected survival probabilities were added in the study of Ting et al., erlotinib became less expensive and survival decreased. This yielded an ICER of $534,903 for afatinib versus erlotinib (incremental costs $7494 and incremental QALY 0.014) [50].

Our results were similar to the cost-effectiveness ratios reported by Chouaid et al. [53] and the National Institute of Health and care Excellence (NICE) [52]. Chouaid et al. [53] assessed the cost-effectiveness of afatinib compared to gefitinib by use of data from the Lux-Lung 7 trial, which resulted in incremental costs of €45,211 per QALY gained. The study by NICE yielded into a cost-effectiveness ratio of £10,076 per QALY gained of afatinib versus erlotinib [52].

However, our study had several limitations. The first limitation was the use of a model-based approach (based on published RCT data), due to a lack of real-world data. Consequently, the results and conclusions of our study are dependent on the validity of the assumptions made in our model. However, various alternative assumptions were assessed through sensitivity analyses, which showed the robustness of our results.

Second, the survival probabilities of gefitinib, erlotinib, afatinib, and osimertinib were estimated by use of the EURTAC trial, which was a trial with predominantly Caucasian population. However, we also included trials with predominantly Asian population in the model, since trials with non-Asian patients for all four TKIs were not available during study period. Although Asian ethnicity is one of the risk factors for EGFR mutations [8], two studies showed no significantly different risk of progression between Asian and non-Asian patients [15, 50]. Thus, use of studies with predominantly Asian population is not expected to bias the efficacy of TKIs. Therefore, to our opinion, the results of our study could be generalised to the Dutch population.

Due to a lack of relevant data on all TKIs, we were not able to perform subgroup analyses, e.g., patients with and without brain metastases. This could be regarded as a limitation, as these analyses might give more insight into the cost-effectiveness of EGFR-TKIs in subgroups [54]. Since brain metastases occur less frequent in patients treated with osimertinib compared to patients treated with gefitinib or erlotinib, it is expected that the QALY gain for osimertinib will increase [19]. Thus, the ICER for this subgroup will be slightly lower compared to the outcomes for the total population. As the occurrence of brain metastases might have a substantial impact on the outcomes, further research on these subgroups is needed.

Furthermore, at the time of our study, the OS results of the FLAURA trial were still immature. Therefore, interim analysis of OS was used in our model. However, the use of final OS results would be more desirable, because it reduces the uncertainty of the model outcomes.

In addition, we assumed that patients treated with the first-line gefitinib, erlotinib, or afatinib all received the same second-line treatments with the same proportions, namely osimertinib (50%) or pemetrexed–cisplatin (50%) and after progression on these second-line treatments, and patients were treated with BSC. Though it may be reasonable that these proportions differ per TKI, we had no data to make such distinctions. Besides that, in reality, patients may also receive other second- or third-line treatments than those included in our model. In the ideal situation, we could fully account for the costs and effects of all second- and third-line treatments used in Dutch clinical practice. However, in the absence of any clear guidance on the second- and third-line treatment strategies after TKI failure [55, 56], we considered our assumption a valid strategy. Scenario analysis also showed a marginal impact of different second-line treatments on the costs. In further research, it is recommended to use real-world data of the first-line and second- and third-line treatment strategy, when it is available.

Furthermore, treatment costs could be overestimated somewhat as we did not adjust for dose reductions. However, adjustment for dose reductions is expected not to have a large impact on the cost-effectiveness results, since the costs related to osimertinib are high anyway. The assumption of no drug wastage is justified, because TKIs are pills and second-line pemetrexed–cisplatin was received by a relatively small proportion of patients, which is expected to have a small amount of drug wastage. The effect on the incremental differences would be negligible. However, it might be more precise when drug wastage is taken into account where relevant.

The clinical effectiveness of osimertinib for patients with EGFR-mutated NSCLC is promising, as it could improve PFS and OS. Moreover, central nervous system (CNS) progression occurred less frequent in patients treated with osimertinib compared to standard-TKI [19]. Besides the substantial clinical relevance, the costs of treating CNS metastases will also be lower for osimertinib versus standard-TKI. Despite these benefits, our results showed that osimertinib could not be regarded as cost-effective compared to all other TKIs. Therefore, it is of great importance to negotiate a lower price for osimertinib.

## Conclusion

This study showed that the cost-effectiveness of afatinib compared to erlotinib is well below the Dutch threshold ratio of €80,000/QALY for treatments in this disease severity group. Osimertinib yielded a better effectiveness compared to afatinib. However, the ICER of osimertinib versus afatinib (€128,343 per QALY gained) appears to be too high given the Dutch threshold. The price of osimertinib should be reduced by 30% to become cost-effective.

## Electronic supplementary material

Below is the link to the electronic supplementary material.

Supplementary material 1 (DOCX 2213 kb)

Supplementary material 2 (DOCX 1718 kb)

Supplementary material 3 (DOCX 25 kb)
