# Supplementary material for: Cost-effectiveness analysis of the first-line EGFR-TKIs in patients with non-small cell lung cancer harbouring EGFR mutations
Source: Eur J Health Econ. 2019 Sep 20;21(1):153–64. doi: 10.1007/s10198-019-01117-3 (PMC7058671; doi:10.1007/s10198-019-01117-3)
Supplement: Supplementary file 1 — Supplementary material 1 (DOCX 2213 kb) [file 10198_2019_1117_MOESM1_ESM.docx]

**Appendix I. Systematic review and NMA**

- 1. ***Search strategy***

A systematic literature search was conducted in three electronic databases (PubMed, EMBASE, and Cochrane Library). Full details on the search strategy and key words can be found in Tables A1-A3. We included articles published from 1 January 2010 up to and including 1 November 2016. The literature search was manually updated in February 2018 to ensure that no relevant studies were missing, as new trials have been published since the last update of our systematic review. Phase IIB/III RCTs that compared the efficacy and toxicity of first-line single EGFR-TKI therapy (including gefitinib, erlotinib, afatinib, and osimertinib) compared to another TKI or standard chemotherapy (platinum-based doublet therapy) in patients with stage IIIB/IV NSCLC harbouring EGFR mutations who were ineligible for surgery or radiotherapy were included. Abstracts, systematic reviews, meta-analyses, and subgroup or post hoc analyses were excluded. We only include English language articles (see Table A4). Literature review and data extraction were conducted by two independent reviewers (MH and CU). Any discrepancies were discussed. Reference lists of published systematic reviews and meta-analyses were checked to ensure that no studies were missed. Quality and risk of bias of all included studies was assessed by using the Cochrane Collaboration’s tool for assessing risk of bias. All included studies had an acceptable quality and low risk of bias (Table A5).

*Table A1. Search strategy PubMed*

|  | **Database PubMed** |
| --- | --- |
|  | **Date of search 10 November 2016** |
|  | **Date range 1 January 2010 - 1 November 2016** |
|  |  |
| 1 | (("afatinib") AND "advanced non small cell lung cancer") AND "egfr mutations" |
| 2 | ((("afatinib") AND "stage 4") AND "non small cell lung cancer") AND "egfr mutations" |
| 3 | (("afatinib") AND "versus") AND "chemotherapy" |
| 4 | ((("afatinib") AND "versus") AND "chemotherapy") AND "first line therapy" |
| 5 | (((("afatinib") AND "versus") AND "chemotherapy") AND "advanced non small cell lung cancer") AND "egfr mutations" |
| 6 | ((((("afatinib") AND "versus") AND "chemotherapy") AND "stage 4") AND "non small cell lung cancer") AND "egfr mutations" |
| 7 | ((("afatinib") AND "versus") AND "chemotherapy") AND "overall survival" |
| 8 | ((("afatinib") AND "versus") AND "chemotherapy") AND "progression free survival" |
| 9 | (("erlotinib") AND "advanced non small cell lung cancer") AND "egfr mutations" |
| 10 | ((("erlotinib") AND "stage 4") AND "non small cell lung cancer") AND "egfr mutations" |
| 11 | (("erlotinib") AND "versus") AND "chemotherapy" |
| 12 | ((("erlotinib") AND "versus") AND "chemotherapy") AND "first line therapy" |
| 13 | (((("erlotinib") AND "versus") AND "chemotherapy") AND "advanced non small cell lung cancer") AND "egfr mutations" |
| 14 | ((((("erlotinib") AND "versus") AND "chemotherapy") AND "stage 4") AND "non small cell lung cancer") AND "egfr mutations" |
| 15 | ((("erlotinib") AND "versus") AND "chemotherapy") AND "overall survival" |
| 16 | ((("erlotinib") AND "versus") AND "chemotherapy") AND "progression free survival" |
| 17 | (("gefitinib") AND "advanced non small cell lung cancer") AND "egfr mutations" |
| 18 | ((("gefitinib") AND "stage 4") AND "non small cell lung cancer") AND "egfr mutations" |
| 19 | (("gefitinib") AND "versus") AND "chemotherapy" |
| 20 | ((("gefitinib") AND "versus") AND "chemotherapy") AND "first line therapy" |
| 21 | (((("gefitinib") AND "versus") AND "chemotherapy") AND "advanced non small cell lung cancer") AND "egfr mutations" |
| 22 | ((((("gefitinib") AND "versus") AND "chemotherapy") AND "stage 4") AND "non small cell lung cancer") AND "egfr mutations" |
| 23 | ((("gefitinib") AND "versus") AND "chemotherapy") AND "overall survival" |
| 24 | ((("gefitinib") AND "versus") AND "chemotherapy") AND "progression free survival" |

*Table A2. Search strategy Embase*

|  | **Database Embase** |
| --- | --- |
|  | **Date of search 21 November 2016** |
|  | **Date range 1 January 2010 - 1 November 2016** |
|  |  |
| 1 | 'afatinib'/exp OR 'afatinib' AND 'advanced non small cell lung cancer' AND 'egfr mutation' AND [2010-2016]/py |
| 2 | 'afatinib'/exp OR 'afatinib' AND 'stage 4' AND 'non small cell lung cancer' AND 'egfr mutation' AND [2010-2016]/py |
| 3 | 'afatinib'/exp OR 'afatinib' AND versus AND 'chemotherapy' AND [2010-2016]/py |
| 4 | 'afatinib'/exp OR 'afatinib' AND versus AND ('chemotherapy'/exp OR 'chemotherapy') AND 'first line therapy' AND [2010-2016]/py |
| 5 | afatinib'/exp OR 'afatinib' AND versus AND ('chemotherapy'/exp OR 'chemotherapy') AND 'advanced non small cell lung cancer' AND 'egfr mutation' AND [2010-2016]/py |
| 6 | 'afatinib'/exp OR 'afatinib' AND versus AND ('chemotherapy'/exp OR 'chemotherapy') AND 'stage 4' AND 'non small cell lung cancer' AND 'egfr mutation' AND [2010-2016]/py |
| 7 | 'afatinib'/exp OR 'afatinib' AND versus AND ('chemotherapy'/exp OR 'chemotherapy') AND 'overall survival' AND [2010-2016]/py |
| 8 | 'afatinib'/exp OR 'afatinib' AND versus AND ('chemotherapy'/exp OR 'chemotherapy') AND 'progression free survival' AND [2010-2016]/py |
| 9 | 'erlotinib'/exp OR 'erlotinib' AND 'advanced non small cell lung cancer' AND 'egfr mutation' AND [2010-2016]/py |
| 10 | 'erlotinib'/exp OR 'erlotinib' AND 'stage 4' AND 'non small cell lung cancer' AND 'egfr mutation' AND [2010-2016]/py |
| 11 | erlotinib'/exp OR 'erlotinib' AND versus AND 'chemotherapy' AND [2010-2016]/py |
| 12 | erlotinib'/exp OR 'erlotinib' AND versus AND ('chemotherapy'/exp OR 'chemotherapy') AND 'first line therapy' AND [2010-2016]/py |
| 13 | 'erlotinib'/exp OR 'erlotinib' AND versus AND ('chemotherapy'/exp OR 'chemotherapy') AND 'advanced non small cell lung cancer' AND 'egfr mutation' AND [2010-2016]/py |
| 14 | 'erlotinib'/exp OR 'erlotinib' AND versus AND ('chemotherapy'/exp OR 'chemotherapy') AND 'stage 4' AND 'non small cell lung cancer' AND 'egfr mutation' AND [2010-2016]/py |
| 15 | 'erlotinib'/exp OR 'erlotinib' AND versus AND ('chemotherapy'/exp OR 'chemotherapy') AND 'overall survival' AND [2010-2016]/py |
| 16 | 'erlotinib'/exp OR 'erlotinib' AND versus AND ('chemotherapy'/exp OR 'chemotherapy') AND 'progression free survival' AND [2010-2016]/py |
| 17 | 'gefitinib' AND 'advanced non small cell lung cancer' AND 'egfr mutation' AND [2010-2016]/py |
| 18 | 'gefitinib'/exp OR 'gefitinib' AND 'stage 4' AND ('non small cell lung cancer'/exp OR 'non small cell lung cancer') AND 'egfr mutation' AND [2010-2016]/py |
| 19 | 'gefitinib' AND versus AND 'chemotherapy' AND [2010-2016]/py |
| 20 | 'gefitinib'/exp OR 'gefitinib' AND versus AND ('chemotherapy'/exp OR 'chemotherapy') AND 'first line therapy' AND [2010-2016]/py |
| 21 | 'gefitinib'/exp OR 'gefitinib' AND versus AND ('chemotherapy'/exp OR 'chemotherapy') AND 'advanced non small cell lung cancer' AND 'egfr mutation' AND [2010-2016]/py |
| 22 | gefitinib'/exp OR 'gefitinib' AND versus AND ('chemotherapy'/exp OR 'chemotherapy') AND 'stage 4' AND 'non small cell lung cancer' AND 'egfr mutation' AND [2010-2016]/py |
| 23 | 'gefitinib'/exp OR 'gefitinib' AND versus AND ('chemotherapy'/exp OR 'chemotherapy') AND ('overall survival'/exp OR 'overall survival') AND [2010-2016]/py |
| 24 | 'gefitinib'/exp OR 'gefitinib' AND versus AND ('chemotherapy'/exp OR 'chemotherapy') AND ('progression free survival'/exp OR 'progression free survival') AND [2010-2016]/py |

*Table A3. Search strategy Cochrane Library*

|  | **Database Cochrane library** |
| --- | --- |
|  | **Date of search 2 December 2016** |
|  | **Date range 1 January 2010 - 1 November 2016** |
|  |  |
| 1 | '"afatinib" and "advanced" and "non small cell lung cancer" and "egfr mutations" , Publication Year from 2010 to 2016 in Trials' |
| 2 | '"afatinib" and "non small cell lung cancer stage IV" and "egfr mutations" , Publication Year from 2010 to 2016' |
| 3 | '"afatinib" and "chemotherapy" , Publication Year from 2010 to 2016 in Trials' |
| 4 | '"afatinib" and "chemotherapy" and "first-line therapy" , Publication Year from 2010 to 2016 in Trials' |
| 5 | '"afatinib" and "chemotherapy" and "advanced" and "non small cell lung cancer" and "egfr mutations" , Publication Year from 2010 to 2016 in Trials' |
| 6 | '"afatinib" and "chemotherapy" and "non small cell lung cancer stage IV" and "egfr mutations" , Publication Year from 2010 to 2016' |
| 7 | '"afatinib" and "chemotherapy" and "overall survival" , Publication Year from 2010 to 2016 in Trials' |
| 8 | '"afatinib" and "chemotherapy" and "progression-free survival" , Publication Year from 2010 to 2016 in Trials |
| 9 | "erlotinib" and "advanced" and "non small cell lung cancer" and "egfr mutations" , Publication Year from 2010 to 2016 in Trials' |
| 10 | "erlotinib" and "non small cell lung cancer stage IV" and "egfr mutations" , Publication Year from 2010 to 2016 in Trials' |
| 11 | '"erlotinib" and "chemotherapy" , Publication Year from 2010 to 2016 in Trials' |
| 12 | '"erlotinib" and "chemotherapy" and "first-line therapy" , Publication Year from 2010 to 2016 in Trials' |
| 13 | "erlotinib" and "chemotherapy" and "advanced" and "non small cell lung cancer" and "egfr mutations" , Publication Year from 2010 to 2016 in Trials' |
| 14 | '"erlotinib" and "chemotherapy" and "non small cell lung cancer stage IV" and "egfr mutations" , Publication Year from 2010 to 2016' |
| 15 | "erlotinib" and "chemotherapy" and "overall survival" , Publication Year from 2010 to 2016 in Trials' |
| 16 | '"erlotinib" and "chemotherapy" and "progression-free survival" , Publication Year from 2010 to 2016 in Trials' |
| 17 | '"gefitinib" and "advanced" and "non small cell lung cancer" and "egfr mutations" , Publication Year from 2010 to 2016 in Trials' |
| 18 | '"gefitinib" and "non small cell lung cancer stage IV" and "egfr mutations" , Publication Year from 2010 to 2016' |
| 19 | '"gefitinib" and "chemotherapy" , Publication Year from 2010 to 2016 in Trials' |
| 20 | '"gefitinib" and "chemotherapy" and "first-line therapy" , Publication Year from 2010 to 2016 in Trials' |
| 21 | "gefitinib" and "chemotherapy" and "advanced" and "non small cell lung cancer" and "egfr mutations" , Publication Year from 2010 to 2016 in Trials' |
| 22 | "gefitinib" and "chemotherapy" and "non small cell lung cancer stage IV" and "egfr mutations" , Publication Year from 2010 to 2016' |
| 23 | '"gefitinib" and "chemotherapy" and "overall survival" , Publication Year from 2010 to 2016 in Trials' |
| 24 | '"gefitinib" and "chemotherapy" and "progression-free survival" , Publication Year from 2010 to 2016 in Trials' |

***
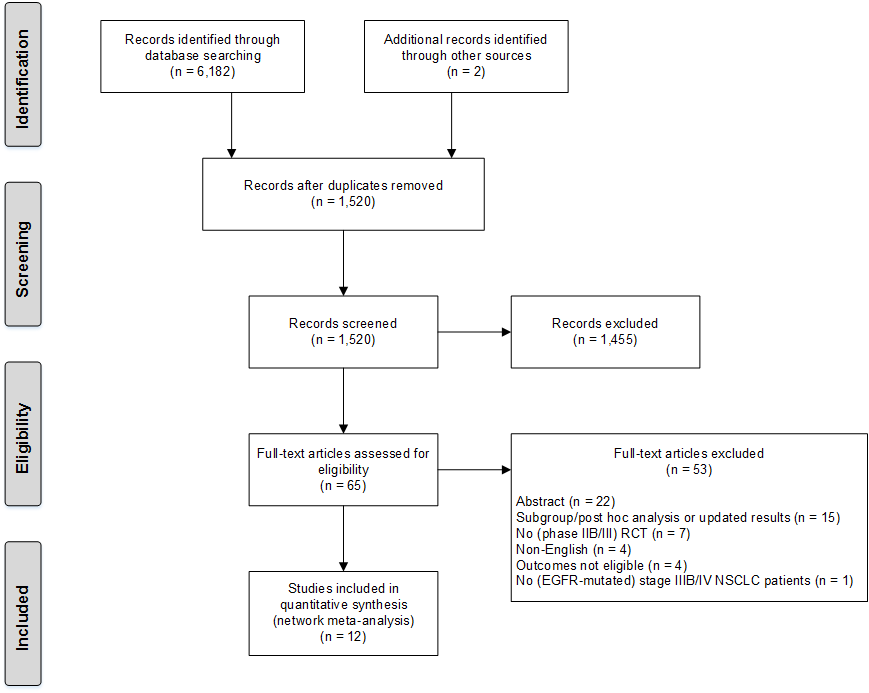
***

*Figure A1. Flow diagram of literature review*

*Table A4. In- and exclusion criteria title/abstract and full-text screening*

| **Title/abstract screening** | | |
| --- | --- | --- |
|  | Exclusion criteria | Total number excluded studies |
| 1 | No first-line therapy | 298 |
| 2 | No (EGFR-mutated) NSCLC study population | 236 |
| 3 | First-line gefitinib, erlotinib, afatinib, or osimertinib not compared to other single TKI or platinum-based doublet therapy | 201 |
| 4 | No (phase IIB/III) RCT | 177 |
| 5 | (Systematic) review/overview | 151 |
| 6 | TKI combination treatment | 145 |
| 7 | Study subject was biomarker/DNA/molecular assessment | 114 |
| 8 | (Network) meta-analysis | 72 |
| 9 | Outcomes not eligible | 37 |
| 10 | NSCLC stage I-IIIA | 17 |
| 11 | Abstract | 7 |
| **Full-text screening** | | |
| 1 | Abstract | 22 |
| 2 | Subgroup/post hoc analysis/updated results | 15 |
| 3 | No (phase IIB/III) RCT | 7 |
| 4 | Non-English | 4 |
| 5 | Outcomes not eligible | 4 |
| 6 | No (EGFR-mutated) stage IIIB/IV NSCLC patients | 1 |

*Table A5. Quality and risk of bias assessment of all included studies*

| **Trial** | **Sequence generation** | **Allocation concealment** | **Blinding of participants** | **Blinding of outcome data** | **Incomplete outcome data** | **Selective reporting** | **Other sources of bias** |
| --- | --- | --- | --- | --- | --- | --- | --- |
| **NEJ002**  (2010) | Low risk | Unclear risk | Unclear risk | Low risk | Low risk | Low risk | Crossover was recommended as second-line treatment |
| **WJTOG 3405** (2010) | Low risk | Low risk | Unclear risk | Low risk | Low risk | Low risk | Crossover was recommended as second-line treatment |
| **IPASS**  (2009/2011) | Low risk | Unclear risk | Unclear risk | Low risk | Low risk | Low risk | Crossover was recommended as second-line treatment |
| **First-SIGNAL**  (2012) | Low risk | Unclear risk | Unclear risk | Low risk | Low risk | Low risk | A maximum of 9 cycles of GP chemotherapy was recommended instead of 4-6 cycles.  Crossover was recommended as second-line treatment |
| **OPTIMAL**  (2011) | Low risk | Low risk | Unclear risk | Low risk | Low risk | Low risk | Crossover was recommended as second-line treatment |
| **EURTAC**  (2012) | Low risk | Unclear risk | Low risk | Low risk | Low risk | Low risk | Crossover was recommended as second-line treatment |
| **ENSURE**  (2015) | Low risk | Unclear risk | Unclear risk | Low risk | Low risk | Low risk | Crossover was recommended as second-line treatment |
| **Lux-Lung 3**  (2013) | Low risk | Low risk | Unclear risk | Low risk | Low risk | Low risk | Crossover was recommended as second-line treatment |
| **Lux-Lung 6**  (2014) | Low risk | Low risk | Unclear risk | Low risk | Low risk | Low risk | Crossover was recommended as second-line treatment |
| **Lux-Lung 7**  (2016) | Low risk | Low risk | Unclear risk | Low risk | Low risk | Low risk | Crossover was recommended as second-line treatment |
| **CTONG0901**  (2017) | Low risk | Unclear risk | Unclear risk | Unclear risk | Low risk | Low risk |  |
| ***FLAURA***  (2018) | Low risk | Unclear risk | Unclear risk | Low risk | Low risk | Low risk | Crossover was recommended as second-line treatment |

***1.2 Methods network meta-analysis***

The formula used to estimate the HR of treatment a versus b, is as follows: $\hat{{HR}_{a,b}}=(e^{{(\delta}_{b}-\delta_{a)}})$. This formula was also used for all other comparisons. Chemotherapy was used as reference treatment in the network $\left( \delta chemo=0 \right)$. Direct and indirect evidence from the RCTs was used to estimate all other $\delta$’s. Convergence was assessed by use of the Brooks-Gelman-Rubin diagnostics, which enabled to determine of the number of burn-in simulations that should be discarded.

Since no separate HRs of osimertinib versus gefitinib and of osimertinib versus erlotinib were reported in the FLAURA trial, the HRs of PFS and OS were assumed to be the same for osimertinib versus gefitinib and for osimertinib versus erlotinib.

***1.3 WinBUGS code***

model{

for(i in 1:ns2) { # LOOP THROUGH 2-ARM STUDIES

# normal likelihood

y[i,2] ~ dnorm(delta[i,2],prec[i,2])

#Deviance contribution for trial i

resdev[i] <- (y[i,2]-delta[i,2])*(y[i,2]-delta[i,2])*prec[i,2]

}

for(i in 1:(ns2)){ # LOOP THROUGH ALL STUDIES

for (k in 2:na[i]) { # LOOP THROUGH ARMS

var[i,k] <- pow(se[i,k],2) # calculate variances

prec[i,k] <- 1/var[i,k] # set precisions

delta[i,k] <- d[t[i,k]] - d[t[i,1]]

}

}

totresdev <- sum(resdev[]) #Total Residual Deviance

d[1]<-0 # treatment effect is zero for reference treatment

# vague priors for treatment effects

for (k in 2:nt){ d[k] ~ dnorm(0,.0001) }

# pairwise ORs and LORs for all possible pair-wise comparisons, if nt>2

for (c in 1:(nt-1)) {

for (k in (c+1):nt) {

HR[c,k] <- exp(d[k] - d[c])

lnHR[c,k] <- (d[k]-d[c])

}

}

# ranking on relative scale

for (k in 1:nt) {

# rk[k] <- nt+1-rank(d[],k) # assumes events are “good”

rk[k] <- rank(d[],k) # assumes events are “bad”

best[k] <- equals(rk[k],1) #calculate probability that treat k is best

# calculates probability that treat k is h-th best

for (h in 1:nt){ prob[h,k] <- equals(rk[k],h) }

}

}

# *** PROGRAM ENDS

##Data

list(ns2=13, nt=5)

t[,1] t[,2] y[,2] se[,2] na[] #Study/Comparison

1 2 -0.71539279 0.190855564 2 # WJTOG3405 / Gef vs Chemo

1 2 -1.133203733 0.157751813 2 # NEJ002 / Gef vs Chemo

1 2 -0.733969175 0.146776568 2 # IPASS / Gef vs Chemo

1 3 -1.832581464 0.24375292 2 # OPTIMAL / Erl vs Chemo

1 2 -0.608806032 0.359274 2 # First-SIGNAL / Gef vs Chemo

1 3 -0.994252273 0.196456179 2 # EURTAC / Erl vs Chemo

1 4 -0.544727175 0.151915487 2 # Lux-Lung 3 / Afa vs Pem

1 4 -1.272965676 0.170364636 2 # Lux-Lung 6 / Afa vs Chemo

1 3 -0.867500568 0.228014764 2 # ENSURE / Erl vs Chemo

2 4 -0.314710745 0.130312659 2 # Lux-Lung 7 / Afa vs Gef

2 3 -0.040821995 0.171216396 2 # CTONG0901 / Erl vs Gef

2 5 -0.798507696 0.117227635 2 # FLAURA / Osi vs Gef

3 5 -0.798507696 0.117227635 2 # FLAURA / Osi vs Erl

END

##Inits

#chain1

list(d=c( NA, 0,0,0,0))

#chain2

list(d=c( NA, 1,1,1,1))

#chain3

list(d=c( NA, 2,2,2,2))

***1.4 Results network meta-analysis***

The characteristics of all included studies are presented in Table A6.

Figure A2 shows the complete network of all included RCTs. Three different chains with 60,000 iterations each were simulated. In each chain, 30,000 iterations were discarded due to a burn-in period. Thus, the results were based on a total sample of 90,000 iterations. The results of the NMA are presented in Table A7.

*Table A6. Characteristics of all included studies*

|  | Trial | Treatment | EGFR patients | Primary end-point | Hazard ratio (95% CI)  PFS OS | |
| --- | --- | --- | --- | --- | --- | --- |
| 1 | ***NEJ002*** | Gefitinib  TC | 114  114 | PFS | 0.30  (0.22-0.41) | 0.887  (0.634-1.241) |
| 2 | ***WJTOG3405*** | Gefitinib  DP | 86  86 | PFS | 0.489  (0.336-0.710) | 1.252  (0.883-1.775) |
| 3 | ***IPASS*** | Gefitinib  TC | 132  129 | OS | 0.48  (0.36-0.64) | 1.00  (0.76-1.33) |
| 4 | ***First-SIGNAL*** | Gefitinib  GP | 26  16 | OS | 0.544  (0.269-1.1) | 1.043  (0.498-2.182) |
| 5 | ***OPTIMAL*** | Erlotinib  GC | 82  72 | PFS | 0.16  (0.10-0.26) | 1.19  (0.83-1.71) |
| 6 | ***EURTAC*** | Erlotinib  CT | 86  87 | PFS | 0.37  (0.25-0.54) | 1.04  (0.65-1.68) |
| 7 | ***ENSURE*** | Erlotinib  GC | 110  107 | PFS | 0.34  (0.22-0.51) | 0.91  (0.63-1.31) |
| 8 | ***Lux-Lung 3*** | Afatinib  AP | 230  115 | PFS | 0.58  (0.43-0.78) | 0.88  (0.66-1.17) |
| 9 | ***Lux-Lung 6*** | Afatinib  GP | 242  122 | PFS | 0.28  (0.20-0.39) | 0.93  (0.72-1.22) |
| 10 | ***Lux-Lung 7*** | Afatinib  Gefitinib | 160  159 | PFS, OS | 0.73  (0.57-0.95) | 0.86  (0.66-1.12) |
| 11 | ***CTONG0901*** | Erlotinib  Gefitinib | 128  128 | PFS | 0.96  (0.69-1.35) | 0.98  (0.67-1.42) |
| 12 | ***FLAURA*** | Osimertinib  Standard TKI | 279  277 | PFS | 0.46  (0.37-0.57) | 0.63  (0.45-0.88) |

AP,cisplatin+pemetrexed; CT,chemotherapy (not specific); DP,cisplatin+docetaxel; GC,carboplatin+gemcitabine; GP,cisplatin+gemcitabine; TC,carboplatin+paclitaxel; CI, confidence interval; N/A, not available.

**
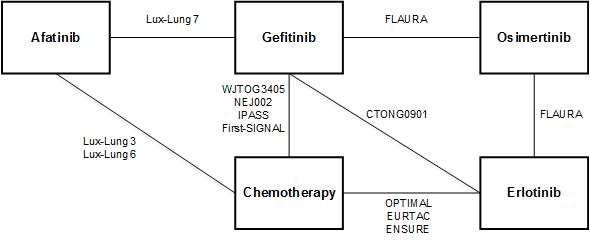
**

*Figure A2. Complete network of all included RCTs*
