# Supplementary material for: Cost-effectiveness analysis of the first-line EGFR-TKIs in patients with non-small cell lung cancer harbouring EGFR mutations
Source: Eur J Health Econ. 2019 Sep 20;21(1):153–64. doi: 10.1007/s10198-019-01117-3 (PMC7058671; doi:10.1007/s10198-019-01117-3)
Supplement: Supplementary file 2 — Supplementary material 2 (DOCX 1718 kb) [file 10198_2019_1117_MOESM2_ESM.docx]

# Appendix II – Methods

## 1.1 Markov model


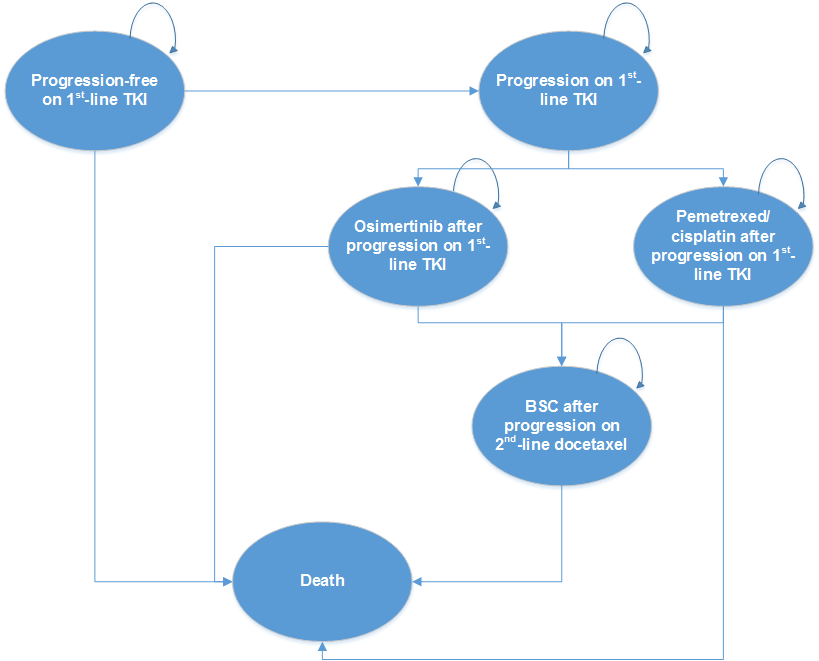


## Figure B1. Schematic diagram of the Markov model

BSC, best supportive care; TKI, tyrosine kinase inhibitor

## 1.2 Survival curves

## Figure B2. OS and PFS curves of first-line gefitinib, erlotinib, afatinib, osimertinib, second-line osimertinib, and pemetrexed/cisplatin

OS, overall survival; pem/cis, pemetrexed/cisplatin; PFS, progression-free survival

## 1.3 Survival probability

## Figure B3. Distributions fitted to Kaplan-Meier curve for OS

## Figure B4. Distributions fitted to Kaplan-Meier curve for PFS

## Table B1. Goodness of fit estimates for OS

|  | AIC | BIC |
| --- | --- | --- |
| Weibull | 510.4461 | 515.3779 |
| Exponential | 510.6748 | 513.1407 |
| Log Logistic | 515.0872 | 520.019 |
| Log Normal | 518.4373 | 523.3691 |

AIC, Akaike Information Criterion; BIC, Bayesian Information Criterion

## Table B2. Goodness of fit estimates for PFS

|  | AIC | BIC |
| --- | --- | --- |
| Weibull | 342.0324 | 346.8459 |
| Log Logistic | 344.0887 | 348.9021 |
| Log Normal | 348.5719 | 353.3853 |
| Exponential | 353.4994 | 355.9061 |

AIC, Akaike Information Criterion; BIC, Bayesian Information Criterion

## Figure B5. Distributions fitted to Kaplan-Meier curve for OS for second-line osimertinib

## Figure B6. Distributions fitted to Kaplan-Meier curve for PFS for second-line osimertinib

## Table B3. Goodness of fit estimates for OS for second-line osimertinib

|  | AIC | BIC |
| --- | --- | --- |
| Exponential | 141.26 | 143.23 |
| Weibull | 143.23 | 147.17 |
| Log Logistic | 143.31 | 147.25 |
| Log Normal | 144.22 | 148.16 |

AIC, Akaike Information Criterion; BIC, Bayesian Information Criterion

## Table B4. Goodness of fit estimates for PFS for second-line osimertinib

|  | AIC | BIC |
| --- | --- | --- |
| Log Normal | 273.41 | 277.59 |
| Log Logistic | 274.08 | 278.27 |
| Exponential | 275.43 | 277.53 |
| Weibull | 277.19 | 281.38 |

AIC, Akaike Information Criterion; BIC, Bayesian Information Criterion

## Figure B7. Distributions fitted to Kaplan-Meier curve for OS for second-line pemetrexed/cisplatin

## Figure B8. Distributions fitted to Kaplan-Meier curve for PFS for second-line pemetrexed/cisplatin

## Table B5. Goodness of fit estimates for OS for second-line pemetrexed/cisplatin

|  | AIC | BIC |
| --- | --- | --- |
| Log Normal | 1028.74 | 1034.60 |
| Weibull | 1030.29 | 1036.16 |
| Log Logistic | 1030.99 | 1036.86 |
| Exponential | 1049.47 | 1052.40 |

AIC, Akaike Information Criterion; BIC, Bayesian Information Criterion

## Table B6. Goodness of fit estimates for PFS for second-line pemetrexed/cisplatin

|  | AIC | BIC |
| --- | --- | --- |
| Log Logistic | 743.14 | 748.83 |
| Log Normal | 745.13 | 750.82 |
| Exponential | 767.83 | 770.67 |
| Weibull | 764.32 | 770.01 |

AIC, Akaike Information Criterion; BIC, Bayesian Information Criterion

## Table B7. Parameters transition probabilities

|  | Value | Reference |
| --- | --- | --- |
| Lambda OS chemotherapy | 0.019 | ^1^ |
| Gamma OS chemotherapy | 1.203 | ^1^ |
| Lambda OS gefitinib | 0.020 |  |
| Gamma OS gefitinib | 1.203 | ^1^ |
| Lambda OS erlotinib | 0.019 |  |
| Gamma OS erlotinib | 1.203 | ^1^ |
| Lambda OS afatinib | 0.017 |  |
| Gamma OS afatinib | 1.203 | ^1^ |
| Lambda OS osimertinib | 0.012 |  |
| Gamma OS osimertinib | 1.203 | ^1^ |
| Intercept OS 2^nd^-line osimertinib | 4.069 | ^2^ |
| Intercept OS 2^nd^-line pem/cis | 2.861 | ^3^ |
| Lambda PFS chemotherapy | 0.073 | ^1^ |
| Gamma PFS chemotherapy | 1.478 | ^1^ |
| Lambda PFS gefitnib | 0.031 |  |
| Gamma PFS gefitinib | 1.478 | ^1^ |
| Lambda PFS erlotinib | 0.026 |  |
| Gamma PFS erlotinib | 1.478 | ^1^ |
| Lambda PFS afatinib | 0.027 |  |
| Gamma PFS afatinib | 1.478 | ^1^ |
| Lambda PFS osimertinib | 0.013 |  |
| Gamma PFS osimertinib | 1.478 | ^1^ |
| Intercept PFS 2^nd^-line osimertinib ^a^ | 2.985 | ^2^ |
| Intercept PFS 2^nd^-line pem/cis | 1.885 | ^3^ |

OS, overall survival; pem/cis, pemetrexed/cisplatin; PFS, progression-free survival

^a^ Transition probability from progression-free to death was estimated by using the following formula: number of patients without progression in previous cycle of second-line treatment*(1 – survival probability OS).

## 1.4 Costs

Patients received oral gefitinib (250mg), erlotinib (150mg), afatinib (40mg), or osimertinib (80mg/day) daily until disease progression or unacceptable toxic effects. Before the start of TKI treatment, tumour tissue was assessed for EGFR-mutations. These testing costs were only applied to the first cycle of the model. Outpatient visits, including laboratory tests, took place every month, both in first-line TKI and second-line osimertinib and pemetrexed/cisplatin treatment <sup>1,2</sup>. Tumour response assessment took place, on average, after every 8 weeks and comprised CT and MRI scan ^1,5-13^. For first-line TKI-treatment, it was assumed that only a very small proportion of patients would receive home care and informal care. These costs were not taken into account for TKIs, because the effect on the ICER will be negligible. Productivity costs were based on a study of Louie et al. ^14^. In this study, productivity loss was measured by using the short form health and labour questionnaire (SF-HLQ) and patient’s productivity costs were estimated by using the friction cost method ^14^.

After progression on gefitinib, erlotinib, or afatinib, patients were tested for T790 M mutations. Patients with T790 M mutation-positive disease (50% of the patients) was treated with 80mg osimertinib on day one of a 30 day cycle. All other patients were treated with 500mg/m^2^ pemetrexed plus 75mg/m^2^ ciplatin after progression on TKI. Costs of pemetrexed/cisplatin were calculated by use of an estimated body surface area of 1.70m² ^15^. Second-line treatment was received until disease progression, unacceptable toxicity or death. Costs for pharmaceutical delivery were added to the drug costs. We assumed that gefitinib, erlotinib, afatinib, and osimertinib were delivered per 30 days each time. For second-line treatment, pharmaceutical costs were taken into account for each cycle ^16^. Travel costs were based on a price per kilometre plus parking costs. According to the Dutch guidelines, a distance of 14 kilometre was used for travelling from a patient’s home to the hospital and back to estimate patient’s travel costs. These costs were applied to the model, both for first-line TKI and second-line treatment. Second-line patients received home care and informal care. Administration costs were added for pemetrexed/cisplatin treatment. We assumed that patients received BSC as third-line treatment after progression on second-line treatment. End-of-life costs comprised costs of the last month of life. These were one-off costs and were added to the health state progression. Severe adverse events were only applied to the first cycle of the model, for both first- and second-line treatment. Costs of adverse events comprised the total costs of the treatment of an adverse event per patient and were multiplied by the probability of each adverse event.

## Table B6. Resource use progression-free and progressive disease health state

|  | Resource use PF (30 days) | Resource use PD (30 days) | Reference |
| --- | --- | --- | --- |
| Costs |  |  |  |
| Gefitinib (250mg/day) | 30.00 | N/A | ^17^ |
| Erlotinib (150mg/day) | 30.00 | N/A | ^17^ |
| Afatinib (40mg/day) | 30.00 | N/A | ^17^ |
| Osimertinib (80mg/day) | 30.00 | N/a | ^17^ |
| Pemetrexed (500mg/m²) | N/A | 1.70 ^a^ | ^17^ |
| Cisplatin (10mg/m²) | N/A | 12.75 ^b^ | ^17^ |
| Best supportive care | N/A | - | ^18^ |
| Mutation test | 1.00 ^c^ | N/A | ^19^ |
| Tumour response assessment | 0.50 | N/A | ^19^ |
| Outpatient visit | 1.00 | N/A | ^16^ |
| Lab tests | 1.00 | 1.00 | ^20^ |
| Concomitant drugs | N/A | 3.00 | ^17^ |
| Administration pem/cis | N/A | 1.00 | ^18^ |
| Home care (per hour) | N/A | 0.15 | ^15^ |
| Informal care (per hour) | N/A | 24.00 | ^15^ |
| Traveling | 1.00 | 1.00 | ^16^ |
| Productivity loss | 1.00* | N/A | ^14^ |
| End-of-life | N/A | 1.00 | ^21,22^ |

N/A, not applicable; pem/cis, pemetrexed/cisplatin; PF, progression-free; PD, progressive disease

^a^ Based on body surface area of 1.70m^2^ and 500mg/m^2^ pemetrexed

^b^ Based on body surface area of 1.70m^2^ and 75mg/m^2^ cisplatin

^c^ One-off costs; only applied to the first cycle of the model

## Table B7. Input parameters for unit costs and probabilities adverse events

|  | Probability AE gefitinib | Probability AE erlotinib | Probability AE afatinib | Probability AE osimertinib | Probability AE 2^nd^-line osimertinib | Probability AE 2^nd^-line pem/cis |
| --- | --- | --- | --- | --- | --- | --- |
| ALT/AST increase | 0.103 | N/A | N/A | N/A | N/A | 0.015 |
| Anaemia | N/A | N/A | N/A | N/A | 0.035 | 0.038 |
| Anorexia | 0.022 | N/A | N/A | N/A | N/A | N/A |
| Asthenia | N/A | N/A | N/A | N/A | 0.025 | 0.030 |
| Decreased appetite | N/A | N/A | 0.018 | 0.025 | N/A | 0.023 |
| Decreased white blood cells | N/A | N/A | N/A | N/A | N/A | 0.091 |
| Diarrhoea | 0.026 | N/A | 0.105 | 0.022 | N/A | N/A |
| Dyspnoea | N/A | N/A | N/A | N/A | 0.025 | 0.023 |
| Fatigue | 0.016 | N/A | 0.021 | N/A | N/A | N/A |
| Febrile neutropenia | N/A | N/A | N/A | N/A | N/A | N/A |
| Leukopenia | N/A | N/A | N/A | N/A | N/A | 0.023 |
| Nausea | N/A | N/A | N/A | N/A | N/A | 0.045 |
| Neuropathy | N/A | N/A | N/A | N/A | N/A | N/A |
| Neutropenia | 0.018 | N/A | N/A | N/A | N/A | 0.053 |
| Paronychia | N/A | N/A | 0.046 | N/A | N/A | N/A |
| Rash | 0.061 | 0.057 | 0.139 | N/A | N/A | N/A |
| Stomatitis | N/A | N/A | 0.064 | N/A | 0.016 | N/A |
| Vomiting | N/A | N/A | N/A | N/A | 0.019 | 0.023 |

N/A, not applicable; pem/cis, pemetrexed/cisplatin; ^1,5-13,23-25^

## References

1. Rosell R, Carcereny E, Gervais R, et al. Erlotinib versus standard chemotherapy as first-line treatment for european patients with advanced EGFR mutation-positive non-small-cell lung cancer (EURTAC): A multicentre, open-label, randomised phase 3 trial. *Lancet Oncol*. 2012;13(3):239-246.

2. Yang JC, Ahn MJ, Kim DW, et al. Osimertinib in pretreated T790M-positive advanced non-small-cell lung cancer: AURA study phase II extension component. *J Clin Oncol*. 2017;35(12):1288-1296.

3. Soria J, Wu Y, Nakagawa K, et al. Gefitinib plus chemotherapy versus placebo plus chemotherapy in *EGFR*-mutation-positive non-small-cell lung cancer after progression on first-line gefitinib (IMPRESS): A phase 3 randomised trial. *Lancet Oncol*. 2015;16(8):990-998.

4. Horgan AM, Bradbury PA, Amir E, et al. An economic analysis of the INTEREST trial, a randomized trial of docetaxel versus gefitinib as second-/third-line therapy in advanced non-small-cell lung cancer. *Ann Oncol*. 2011:1-7.

5. Mitsudomi T, Morita S, Yatabe Y, et al. Gefitinib versus cisplatin plus docetaxel in patients with non-small-cell lung cancer harbouring mutations of the epidermal growth factor receptor (WJTOG3405): An open label, randomised phase 3 trial. *Lancet Oncol*. 2010;11(2):121-128.

6. Zhou C, Wu Y, Chen G, et al. Erlotinib versus chemotherapy as first-line treatment for patients with advanced EGFR mutation-positive non-small-cell lung cancer (OPTIMAL, CTONG-0802): A multicentre, open-label, randomised, phase 3 study. *Lancet Oncol*. 2011;12(8):735-742.

7. Wu Y, Zhou C, Hu C, et al. Afatinib versus cisplatin plus gemcitabine for first-line treatment of asian patients with advanced non-small-cell lung cancer harbouring EGFR mutations (LUX-lung 6): An open-label, randomised phase 3 trial. *Lancet Oncol*. 2014;15(2):213-222.

8. Han J, Park K, Kim S, et al. First-SIGNAL: First-line single-agent iressa versus gemcitabine and cisplatin trial in never-smokers with adenocarcinoma of the lung. *J Clin Oncol*. 2012;30(10):1122-1128.

9. Maemondo M, Inoue A, Kobayashi K, et al. Gefitinib or chemotherapy for Non–Small-cell lung cancer with mutated EGFR. *N Engl J Med*. 2010;362(25):2380-2388.

10. Mok TS, Wu Y, Thongprasert S, et al. Gefitinib or Carboplatin–Paclitaxel in pulmonary adenocarcinoma. *N Engl J Med*. 2009;361(10):947-957.

11. Park K, Tan E, O'Byrne K, et al. Afatinib versus gefitinib as first-line treatment of patients with *EGFR* mutation-positive non-small-cell lung cancer (LUX-lung 7): A phase 2B, open-label, randomised controlled trial. *The Lancet Oncology*. 2016;17(5):577-589.

12. Sequist LV, Yang JC, Yamamoto N, et al. Phase III study of afatinib or cisplatin plus pemetrexed in patients with metastatic lung adenocarcinoma with EGFR mutations. *J Clin Oncol*. 2013;31(27):3327-3334.

13. Wu YL, Zhou C, Liam CK, et al. First-line erlotinib versus gemcitabine/cisplatin in patients with advanced EGFR mutation-positive non-small-cell lung cancer: Analyses from the phase III, randomized, open-label, ENSURE study. *Ann Oncol*. 2015;26(9):1883-1889.

14. Louie AV, van Werkhoven E, Chen H, et al. Patient reported outcomes following stereotactic ablative radiotherapy or surgery for stage IA non-small-cell lung cancer: Results from the ROSEL multicenter randomized trial. *Radiotherapy and Oncology*. 2015;117(1):44-48.

15. Uyl-de Groot CA, Al MJ, Zaim R. *Nivolumab (opdivo) bij gevorderd plaveiselcarcinoom van de long. kosten-effectiviteit en op waarde gebaseerde prijsbenchmarks.* Rotterdam, The Netherlands: iBMG; 2015.

16. Hakkaart-van Roijen L, van der Linden N, Bouwmans CAM, Kanters T, Tan SS. Costing manual [in dutch: Kostenhandleiding]. . 2016.

17. Dutch National Health Care Institute (ZIN). Drug costs [in dutch: Medicijnkosten]. <http://www.medicijnkosten.nl/>. Updated 2016. Accessed March, 2016.

18. Pompen M, Gok M, Novák A, et al. Direct costs associated with the disease management of patients with unresectable advanced non-small-cell lung cancer in the netherlands. *Lung Cancer*. 2009;64(1):110-116.

19. Dutch Healthcare Authority (NZa). DBC tariff application [in dutch: DBC zorgproducten tariefapplicatie]. <http://dbc-zorgproducten-tarieven.nza.nl/nzaZpTarief/Welkom.aspx>. Updated 2016. Accessed March, 2016.

20. van der Linden N, Bongers ML, Coupé VMH, et al. Costs of non-small cell lung cancer in the netherlands. *Lung Cancer*. 2016;91:79-88.

21. Bekelman JE, Halpern SD, Blankart C, al e. Comparison of site of death, health care utilization, and hospital expenditures for patients dying with cancer in 7 developed countries. *JAMA*. 2016;315(3):272-283.

22. Polder JJ, Barendregt JJ, van Oers H. Health care costs in the last year of life—The dutch experience. *Soc Sci Med*. 2006;63(7):1720-1731.

23. Yang JJ, Zhou Q, Yan HH, et al. A phase III randomised controlled trial of erlotinib vs gefitinib in advanced non-small cell lung cancer with EGFR mutations. *Br J Cancer*. 2017(116):568-574.

24. Soria JC, Ohe Y, Vansteenkiste J, et al. Osimertinib in untreated EGFR-mutated advanced non-small-cell lung cancer. *N Engl J Med*. 2018;378(2):113-125.

25. Garon EB, Ciuleanu T, Arrieta O, et al. Ramucirumab plus docetaxel versus placebo plus docetaxel for second-line treatment of stage IV non-small-cell lung cancer after disease progression on platinum-based therapy (REVEL): A multicentre, double-blind, randomised phase 3 trial. *Lancet*. 2014;384(9944):665-673.
