# Supplementary material for: Cost-effectiveness analysis of the first-line EGFR-TKIs in patients with non-small cell lung cancer harbouring EGFR mutations
Source: Eur J Health Econ. 2019 Sep 20;21(1):153–64. doi: 10.1007/s10198-019-01117-3 (PMC7058671; doi:10.1007/s10198-019-01117-3)
Supplement: Supplementary file 3 — Supplementary material 3 (DOCX 25 kb) [file 10198_2019_1117_MOESM3_ESM.docx]

# Appendix III – Results

**Table C1. Cost-effectiveness estimates of all comparisons**

| **Comparison** | **Incremental costs (€)** | **Incremental LYs** | **Incremental QALYs** | **ICER** (€) |
| --- | --- | --- | --- | --- |
| Erlotinib - gefitinib | -1,854 | 0.03 | 0.03 | -68,542 |
| Afatinib - gefitinib | 3,529 | 0.23 | 0.16 | 22,514 |
| Afatinib - erlotinib | 5,383 | 0.20 | 0.13 | 41,504 |
| Osimertinib – afatinib | 62,936 | 0.68 | 0.49 | 129,075 |
| Osimertinib – erlotinib | 68,319 | 0.88 | 0.62 | 110,676 |
| Osimertinib - gefitinib | 66,465 | 0.91 | 0.64 | 103,152 |

ICER, incremental cost-effectiveness ratio

**Table C2. Results scenario analyses**

|  | Incremental costs (€) | Incremental LYs | Incremental QALYs | ICER (€) |
| --- | --- | --- | --- | --- |
| Log logistic function  Erlotinib – gefitinib  Afatinib – erlotinib  Osimertinib – afatinib | -1,440  2,607  22,372 | 0.02  0.12  0.40 | 0.02  0.08  0.28 | -94,805  33,847  81,158 |
| Survival curves based on Lux-Lung 6  Erlotinib – gefitinib  Afatinib – erlotinib  Osimertinib – afatinib | -1,955  4,296  37,531 | 0.03  0.18  0.59 | 0.02  0.12  0.42 | -85,930  37,289  90,013 |
| Second-line docetaxel treatment  Erlotinib – gefitinib  Afatinib – erlotinib  Osimertinib – afatinib | -1,607  5,149  62,045 | 0.03  0.20  0.68 | 0.03  0.13  0.49 | -59,411  39,650  126,900 |
| Price reduction of 30% of osimertinib being cost-effective  Osimertinib – afatinib | 36,275 | 0.68 | 0.49 | 74,396 |

ICER, incremental cost-effectiveness ratio

Figure C1. Tornado diagram of the ICER of gefitinib vs. erlotinib

Figure C2. Tornado diagram of the ICER of afatinib vs. erlotinib
